# Nonsurgical Management of a Large Periapical Lesion Following Instrument Retrieval From the Apical Third: A Case Report With a Three-Year Follow-Up

**DOI:** 10.7759/cureus.24995

**Published:** 2022-05-14

**Authors:** Sudha Yadav, Ruchika R Nawal, Sangeeta Talwar

**Affiliations:** 1 Conservative Dentistry and Endodontics, Maulana Azad Institute of Dental Sciences, New Delhi, IND

**Keywords:** long term follow-up, nonsurgical healing, ultrasonics, apical third, fractured instrument

## Abstract

Instrument separation in the apical third of the tooth, which is associated with a large periapical lesion, presents an arduous task for the clinician. This case report presents nonsurgical endodontic management of a maxillary central incisor associated with a large periapical lesion and a separated instrument in the apical third of the root canal. A 28-year-old male patient presented with pain and labial swelling in the left maxillary central incisor region. Pulp sensibility testing showed no response. The radiograph revealed the presence of a separated instrument in the apical third of the root canal and periapical radiolucency. A diagnosis of previously initiated therapy with acute apical abscess was made. In the first visit, instrument retrieval was done using ProUltra Endo tips (Dentsply Sirona, York, Pennsylvania) under a dental operating microscope. In the subsequent visit, obturation was done as the patient was asymptomatic. The patient was recalled for follow-up at six, 12, 18, 24, and 36 months. Complete healing of the periapical tissues was evident on the radiograph, and the tooth remained functional for the entire follow-up period of three years. The successful outcome seen in this case shows that even large periapical lesions can be managed conservatively by nonsurgical endodontic treatment

## Introduction

Instrument fracture can be a frustrating experience for the clinician and patient as it hinders the biomechanical preparation of the root canal system, thereby reducing the ultimate success of endodontic treatment [1]. Despite the advancements made in the metallurgy of endodontic rotary instruments, instrument fracture remains a major concern. The incidence of instrument separation has increased with the introduction of nickel-titanium (NiTi) rotary instruments (1.3% -10%) as compared to stainless steel files (0.25% - 6%) [2,3]. The separated instrument can potentially reduce the success rate of endodontic therapy, particularly when the tooth is associated with a periapical lesion [1]. Several techniques and kits have been designed for retrieval of the fractured instruments, out of which ultrasonics is quite effective [4]. This case report presents the successful retrieval of a separated instrument from the apical third of the maxillary central incisor using ultrasonics and healing of the associated periapical lesion following the nonsurgical endodontic treatment.

## Case presentation

**Figure 1:**
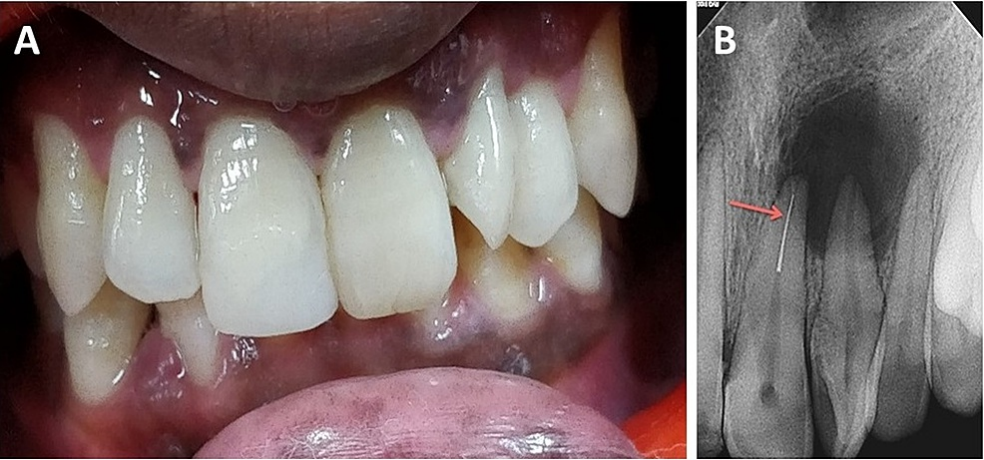
Intraoral photograph and radiograph A: Intraoral photograph showing slightly discolored left central incisor. A well-formed, rotated supernumerary tooth can be seen between the left central and lateral incisor. B: Radiograph showing the separated instrument in the apical third of central incisor (depicted by arrow). Large periapical lesions can be seen associated with central incisor and supernumerary tooth.

A 28-year-old male patient reported to the department of conservative dentistry and endodontics of a tertiary care hospital with a chief complaint of pain and swelling in the left maxillary incisor region. Extra-oral tissues appeared normal. On intra-oral examination, tooth #9 was tender on percussion, for which endodontic treatment was initiated by a private practitioner four months back. A supernumerary tooth (ST) was present between the left maxillary central and lateral incisor (Figure 1A). ST was rotated and well-formed resembling maxillary central incisor morphologically. A fluctuant swelling was present in the labial vestibule corresponding to tooth #9. The patient’s overall periodontal health and occlusion were satisfactory.

On vitality testing using a cold test (Endo Ice; Hygenic, Akron, Ohio) and electric pulp test (Analytic Technology, Leederville, Australia), no response was elicited wrt tooth #9 and ST. A normal response was seen wrt tooth #8 and tooth #10. Radiographic examination revealed a separated endodontic instrument measuring 5.5 cm in the left maxillary central incisor present in the apical third of the root canal. Large periapical radiolucency (dimensions 11 ×11mm approximately) was associated with maxillary central incisor and supernumerary tooth (Figure 1B). Based on the clinical and radiographic presentation, a diagnosis of previously initiated therapy with acute apical abscess was made. The patient was informed about the separated instrument present in the tooth, and treatment options were discussed. The decision to attempt instrument retrieval followed by nonsurgical endodontic treatment was made. Informed, valid consent was obtained from the patient.

**Figure 2:**
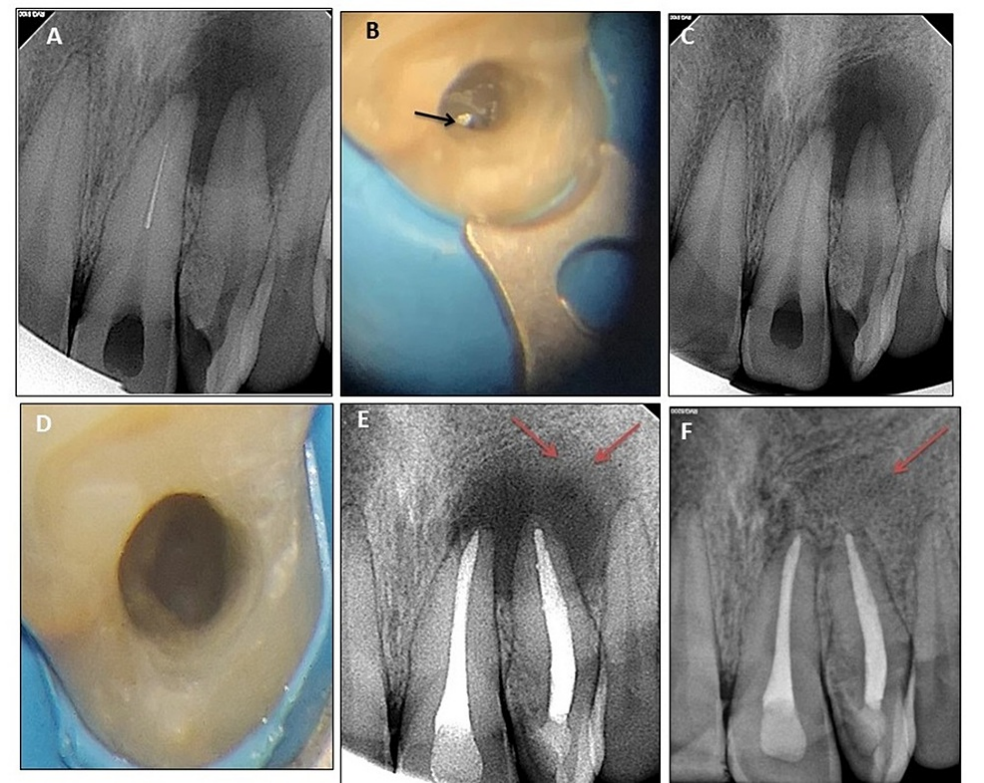
Intraoperative radiographs and dental operating microscope images (A) Instrument retrieval was attempted using ultrasonics and instrument displacement in the coronal direction. (B) Separated instrument visualized under dental operating microscope (shown with arrow). (C) Radiograph after Instrument retrieval. (D) Empty canal lumen seen after instrument retrieval under dental operating microscope. (E) Six-month recall radiograph showing the beginning of osseous healing peripherally (marked with arrows). (F) Three-year recall radiograph showing complete osseous healing (marked with arrow).

In the first appointment, access cavity preparation was modified wrt tooth #9 and the supernumerary tooth (ST) after rubber dam isolation under a surgical operating microscope (Proergo; Zeiss, Oberkochen, Germany). Modified Gates Glidden drill no. 3 (Dentsply Maillefer, Ballaigues, Switzerland) was used at a speed of 800 rpm to prepare a staging platform wrt tooth #9. Gates Glidden drill was modified by cutting it perpendicular to its long axis at its greatest diameter. The fractured instrument was visible under the operating microscope at this stage. Ultrasonic tip ProUltra ENDO No 8 (Dentsply Sirona, York, Pennsylvania) was positioned between the canal wall and fractured instrument and activated at the lowest power setting (Spartan Ultrasonic Endo J15; Obtura Spartan Endodontics, Algonquin, Illinois) by moving it in the counterclockwise direction in dry conditions. The instrument was loosened in the canal and moved in the coronal direction, as seen on the intraoperative radiograph (Figure 2A, 2B). However, due to the long length of the fractured instrument, it didn’t float out of the canal. After several attempts, the separated fragment was bypassed using a 20# K-file (Dentsply Maillefer, Ballaigues, Switzerland). Following this, a 20# H-file was introduced in the canal up to working length and was withdrawn allowing the retrieval of the fractured fragment (Figure 2C, 2D). On inspection, the fractured fragment was found to be NiTi rotary file. Straw-colored discharge was evident from tooth #9 on instrument retrieval.

Working length was assessed radiographically for both teeth. Biomechanical preparation was done using hand K files using the step-back technique for tooth #9 and ST. Apical preparation was done till 60# K-file and 45# K-file for tooth #9 and ST, respectively. Irrigation was done using 5.25% sodium hypochlorite (NaOCl) (Novo Dental Product, Mumbai, India), followed by 17% ethylenediaminetetraacetic acid (EDTA; Prevest Denpro Limited Digiana, Jammu, India). Canals were rinsed with normal saline in the end. The root canal was dried with sterile paper points, and calcium hydroxide dressing was placed in the canal space. The access cavity was sealed with a 3mm thick provisional restoration material (Coltosol F; Coltene, Altstätten, Switzerland), and the patient was recalled after two weeks. 

The patient was completely asymptomatic on the recall visit. Provisional restoration was removed using #2 diamond round bur. Copious irrigation with 5.25% NaOCl followed by 17% EDTA was done. A final rinse with normal saline was done, and the canal was dried with paper points. Obturation was done with AH Plus Sealer and Gutta-percha (Dentsply Maillefer, Ballaigues, Switzerland) using the lateral condensation technique. The access cavity was restored with nano-hybrid composite resin (Filtek Z250 XT; 3M ESPE, Seefeld, Germany).

The patient was regularly followed up. At the three-year recall visit, the patient was asymptomatic, and both teeth were functional. Osseous healing was evident peripherally on a six-month recall (Figure 2E). Complete healing was seen on the three-years recall visit, and the periapical area showed a normal trabecular pattern (Figure 2F).

## Discussion

Teeth presenting with concomitant large periapical lesion and a fractured instrument in the apical third fall in the category of complex endodontic cases and often requires surgical intervention. However, surgical intervention should be done only when conservative treatment options fail. It has been observed that up to 94.4% of periapical lesions show partial or complete healing when managed with a conservative approach using nonsurgical endodontic therapy [5,6]. In our case as well, the nonsurgical endodontic treatment proved effective.

Another major issue in the presented case was the presence of a separated instrument which is one of the most alarming complications occurring during endodontic treatment. The several attributing factors for instrument fracture are repeated usage of the instruments, instrument design, degree of canal curvature, and operator’s experience [7-9]. However, in the present case, the cause of fracture was difficult to determine as the treatment was initiated by a private practitioner. On inspection, a separated instrument was found to be a NiTi rotary file. As the involved tooth had a single straight canal, presumably, the instrument fracture can be attributed to over-usage.

When a patient presents with a separated instrument, a skilled clinician should always weigh the advantages and disadvantages of attempting instrument retrieval. Lin et al. designed a statistical model to help clinicians in decision-making and concluded that the two most important factors determining the success of instrument retrieval are the depth of separated instrument, and canal curvature [10]. In the presented case, all the factors were favorable. First, the involved tooth was an anterior tooth that has a high success rate of instrument retrieval (81.8%). Secondly, the root canal was straight, and the depth of the separated instrument was less due to the long length of the instrument fragment. Furthermore, the success rate of teeth associated with a periapical lesion is reduced from 89% to 47% in the presence of a periapical lesion as compared to vital teeth with no periapical lesion [11]. Hence it becomes extremely important to either retrieve or bypass the separated instrument in the presence of an associated periapical lesion. In the presented case, the tooth was associated with a large periapical lesion, hence the decision to attempt instrument retrieval.

Attempts to remove an apically located fragment can result in excessive removal of dentin and perforate or weaken the root making it susceptible to fracture [12]. It has been shown that instrument retrieval reduces the root strength by 30-40% [13]. However, in the presented case, the separated instrument was retrieved from the maxillary central incisor, which is easily accessible and has straight roots reducing the chances of any complications. Also, ProUltra Endo tip size 8 was used to minimize the loss of radicular dentin as it has a small diameter and longer length, which aids in reaching an inaccessible apical third of the canal system.

Proper illumination and magnification are the keys to successful instrument retrieval, particularly when the separated instrument is present in the apical third. The separated instrument needs to be carefully located under a dental operating microscope for precise removal of radicular dentin by ultrasonics. The instrument retrieval rate is reported to be as high as 95% with ultrasonics [14]. While using ultrasonics for instrument retrieval, it is important to activate it only when it is in contact with root dentin; otherwise, it might shred or push the separated instrument apically. It has been documented in the literature as well that a longer instrument is easier to remove as compared to a smaller one [15]. Hence it is critical to avoid secondary fracture of the separated fracture as it can further complicate the treatment.

One of the potential drawbacks of the presented case is that cone beam computed tomography (CBCT) was not done postoperatively to confirm the healing of periapical lesion three-dimensionally. However, considering the socio-economic status as well as the clinical status of the patient, CBCT was not done.

## Conclusions

Instrument retrieval is critical for the success of endodontic treatment, especially when the tooth is associated with a periapical lesion. This case report demonstrates a successful retrieval of a separated instrument from the apical third of the root canal with minimal damage to the radicular dentine, which was possible due to the application of extremely fine ultrasonic tips under a dental operating microscope. Also, the use of an advanced armamentarium can reduce the risk of mishaps or procedural errors while managing such complicated cases. 
